# Monitoring voltage fluctuations of intracellular membranes

**DOI:** 10.1038/s41598-018-25083-7

**Published:** 2018-05-02

**Authors:** Masoud Sepehri Rad, Lawrence B. Cohen, Oliver Braubach, Bradley J. Baker

**Affiliations:** 1 Center for Functional Connectomics, Brain Science Institute, Korea Institute of Science and Technology (KIST), Seoul, 02792 Korea; 2 Department of Cellular and Molecular Physiology, Yale University School of Medicine, New Haven, CT 06520 USA; 3 Department of Neuroscience, Korea University of Science and Technology, Daejeon, 34113 South Korea

## Abstract

In eukaryotic cells, the endoplasmic reticulum (ER) is the largest continuous membrane-enclosed network which surrounds a single lumen. Using a new genetically encoded voltage indicator (GEVI), we applied the patch clamp technique to cultured HEK293 cells and neurons and found that there is a very fast electrical interaction between the plasma membrane and internal membrane(s). This discovery suggests a novel mechanism for interaction between the external membrane and internal membranes as well as mechanisms for interactions between the various internal membranes. The ER may transfer electrical signals between the plasma membrane and other internal organelles. The internal membrane optical signal is reversed in polarity but has a time course similar to that of the plasma membrane signal. The optical signal of the GEVI in the plasma membrane is consistent from trial to trial. However, the internal signal decreases in size with repeated trials suggesting that the electrical coupling is degrading and/or the resistance of the internal membrane is decaying.

## Introduction

The internal workings of the eukaryotic cell continue to astound. The ability of fluorescently tagged proteins to monitor the compartmentalization of the organelle interactome provides new insights into how the cell handles environmental conditions including stress^1^. A central player in the organelle interactome is the ER. The ER contacts every other organelle in the cell including the nucleus and plasma membrane^2–5^. This central role of the ER also makes it the stress center of the cell responding to unfolded protein accumulation (unfolded protein response – UPR)^6,7^ as well as nutrient starvation and oxidative stress (integrated stress response - ISR)^8^.

Internal membranes involved in the compartmentalization of the cell may exhibit voltage changes^9^. In addition to the classical biochemical signaling cascades, electrical signals have also been postulated to send information from the cell’s exterior to the nucleus^10^. Such a mechanism has remained speculative since the membrane potential of the ER cannot be directly measured by electrodes. Here we report the surreptitious trafficking of a GEVI that demonstrates the ability of electrical signals at the plasma membrane to affect the voltage of internal membranes.

Internal expression of plasma membrane proteins is usually a problem. For nearly a decade it was the bane of GEVIs^11^. GEVIs are fluorescent proteins which yield optical signals in response to changes in membrane potential. Using the voltage-sensing domain from a voltage-gated potassium channel, Siegel and Isacoff developed the archetype, FlaSh^12^. Using confocal microscopy and a voltage sensitive dye as a surface marker, Baker *et al*.^11^ showed that FlaSh and two other first generation GEVIs were predominantly expressed intracellularly in mammalian cells probably due to protein misfolding. The protein was still fluorescent but did not traffic to the plasma membrane. As a result, no detectable voltage-dependent, optical signals were measured in mammalian cells with these sensors. This deficiency was overcome by using the voltage-sensing domain from the *Ciona intestinalis* voltage-sensing phosphatase^13^.

In this paper we introduce mutations to the loop regions of the Voltage Sensing Domain (VSD) of an ArcLight-type GEVI^14^ resulting in a distinct intracellular optical signal that responds to changes in the plasma membrane potential. The internal signal for this GEVI, Aahn (Korean for inside), is always accompanied by an opposite polarity signal in the plasma membrane enabling simultaneous monitoring of internal membrane potentials in about 20% of the transfected cells. The other 80% of Aahn expressing cells do not exhibit enough internal expression to overcome the reverse polarity of the optical signal from the plasma membrane. The possibility that the other 80% of the cells do not experience a change in internal membrane potentials is real but relative low given that high intracellular fluorescence is an excellent predictor for observation of an internal signal. Regardless, this result indicates that an internal population of the probe is folded correctly, is functional, and demonstrates that alterations in the plasma membrane potential can affect the potential of internal membranes. This internal signal was also observed in neurons suggesting that this phenomenon may be present in many if not all cell types.

## Results

### ArcLight mutations: External and internal optical signals from HEK293 cells and hippocampal neurons

**Figure 1:**
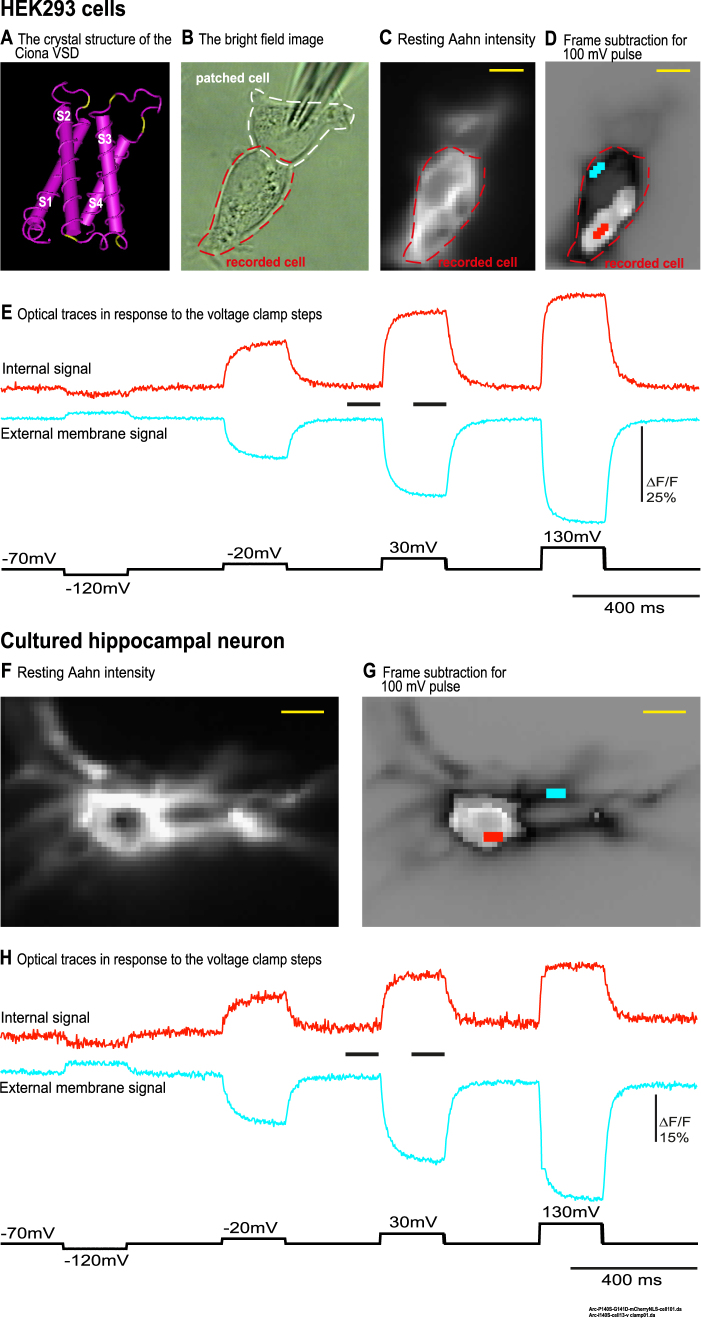
The internal membrane has an electrical interaction with plasma membrane in HEK293 cells and hippocampal neurons. HEK 293 cells and neurons expressing Aahn were voltage-clamped. Unless otherwise indicated, the holding potential was −70 mV. In this and subsequent figures the pulse protocol is shown in the black trace. (**A**) The crystal structure of the *Ciona* voltage-sensing domain. The yellow residues indicate the location of the mutations that yielded probes which have an internal signal distinct from the external, plasma membrane signal. (**B**) The bright field image of HEK293 cells. In this and subsequent figures the white and red outlines indicate the patched cell and recorded cell respectively. (**C**) The resting light fluorescence image of the Aahn expression. (**D**) The frame subtraction image of the HEK293 cells in response to the 100 mV depolarization pulse (white and black are internal membrane and plasma membrane optical signals respectively). In this figure and Figs 2, 3 and 4, 50 frames at the time indicated by the first black bar between the two traces were subtracted from the 50 frames indicated by the black bar during the voltage step. (**E**) The average of first 4 trials observed in the plasma membrane (blue trace) and internal membrane (red trace) without temporal filtering. (**F**) The resting light fluorescence image of a hippocampal neuron expressing Aahn. (**G**) The frame subtraction image of the neuron in response to the 100 mV depolarization pulse. (**H**) The red and blue traces indicate the optical signals (average of first 4 trials) from internal membrane (red trace) and the plasma membrane (blue trace) respectively without temporal filtering. Images were recorded at a frame rate of 500 fps. The size bars represent 10 μm.

With the aim of adjusting the voltage-sensitivity of GEVIs, over 180 unique voltage sensing domains from voltage-gated sodium channels (Na_v_) were aligned to identify highly conserved amino acids in the cytoplasmic and extracellular loops connecting the transmembrane segments. Conserved polar amino acids were found at multiple α-helix/loop junctions in the voltage sensing domain. A similar conservation of polar amino acids was found in the voltage sensing domain of the voltage-sensing phosphatase gene family. Remarkably, some of the mutations in the α-helix/loop junctions in the voltage-sensing domain ([I140S,K],[Y172K,R,S],[N180A],[D204E,K,Y], and [D213A,S,T,Y]) of the GEVI, CC1^15^, yielded probes which have an internal signal distinct from the external, plasma membrane signal. In Fig. 1A, the voltage sensing domain is shown consisting of the four transmembrane segments, S1-S4. The yellow regions indicate the location of these mutations that generated an internal signal. These data suggest that some mutations in the intra/extracellular loop regions of the VSD behave like a new motif for expression in intracellular membranes. However, this novel mechanism does not seem to inhibit the expression of the protein in the plasma membrane. Introduction of two mutations near the external transmembrane/loop junction of S1 (P140S and G141D) into the GEVI, ArcLight, resulted in a new probe, Aahn, capable of optically monitoring both plasma membrane and internal membrane potentials. Figure 1 illustrates the opposite signed fluorescence signals in response to manipulating the plasma membrane potential. The plasma membrane fluorescence gets dimmer (blue trace) during depolarization steps while the internal signal (red trace) gets brighter when expressed in HEK cells. Similarly, Fig. 1 also illustrates a similar opposite signed fluorescence response in a cultured neuron. Since the internal signal is reversed compared to the plasma membrane signal, we suppose either that when the plasma membrane is depolarized, an internal membrane is hyperpolarized, or that the GEVI in the internal membrane is reversed in direction.

HEK293 cells are connected to each other electrically via gap junctions. To avoid physical contact between the patch electrode and internal membranes, a neighboring cell was patched and voltage clamped (indirect patch clamping, e.g., Fig. 1B). Patching and recording from the same cell is designated as direct patch clamping. Direct patch clamping was used for neurons. We carried out 4 or 6 repetitions with four trials in each repetition. Unless indicated, the results come from the average of the four trials in the first of the repetitions.

#### Frame subtraction images identify the location of a distinct, internal, voltage signal

**Figure 2:**
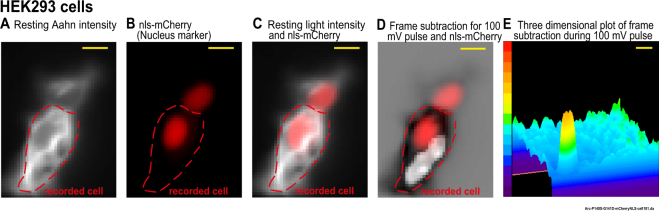
The overlap between nucleus marker and the internal signal area in an HEK293 cell. The region of the internal signal and the nuclear marker do not overlap. HEK 293 cells expressing Aahn and nls-mCherry were voltage-clamped. (**A**) The resting light fluorescence image of Aahn expression. (**B**) A red florescent protein (nls-mCherry) was used as a marker for nuclei. (**C**) The merge of resting light image of the HEK293 cells expressing Aahn (white) and nls-mCherry (red). (**D**) Merge of frame subtraction and nls-mCherry (red) (from the same HEK 293 cell as shown in Fig. 1). (**E**) The 3 dimensional plot of the frame subtraction during the 100 mV depolarization pulse filtered using two iterations of a low-pass spatial filter (3 × 3 Mean). The color scale indicates the light intensity with the red and purple indicating the brightest and the darkest pixels. The size bars represent 10 μm.

**Figure 3:**
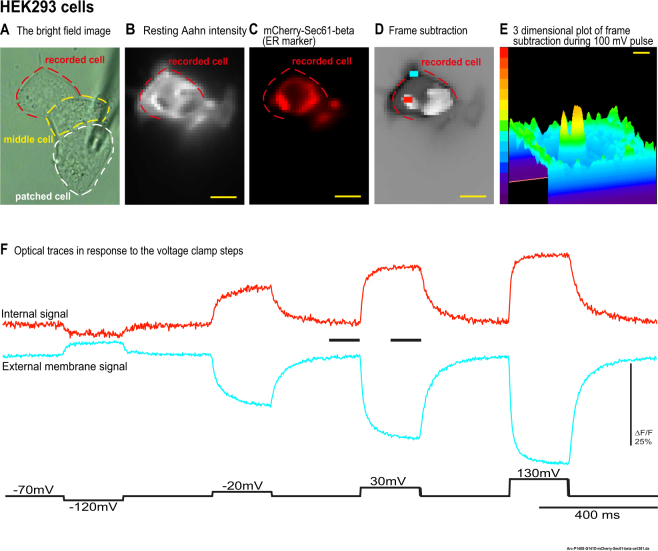
The overlap between an ER marker and the internal signal area in HEK293 cells. HEK 293 cells co-expressing Aahn and mCherry-Sec. 61-beta were voltage-clamped. (**A**) The bright field image of the HEK293 cells. (**B**) The resting light image of the Aahn expression. (**C**) A red florescence protein (mCherry-Sec. 61-beta) was used as a marker for ER. (**D**) The frame subtraction image of the HEK293 cells in response to the 100 mV depolarization pulse (white indicates the internal signal and black indicates the external membrane optical signal). There is a substantial overlap between the area of the internal signal and the ER fluorescence. (**E**) The three dimensional plot of frame subtraction during the 100 mV depolarization pulse (**F**) The red and blue traces are representative fluorescent signals (average of first 4 trials) from internal membrane (red trace) and the plasma membrane (blue trace) respectively without temporal filtering. Images were recorded at a frame rate of 500 fps. Size bars represent 10 μm.

**Figure 4:**
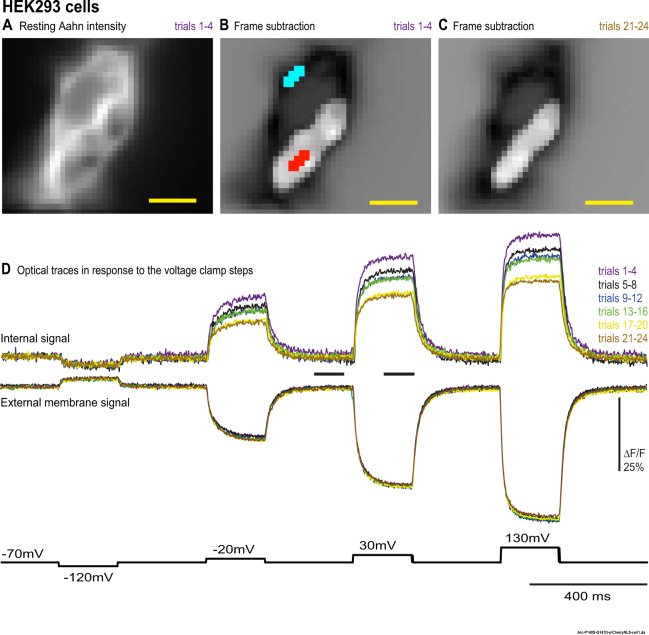
The internal optical signal size decreases during repeated patch clamp measurements in HEK293 cells. A HEK 293 cell (same cell that was illustrated in Figs 1 and 2) expressing Aahn was voltage-clamped. The internal signal, but not the external signal, decreases with repeated trials. (**A**) The resting light image of the Aahn expression. (**B**,**C**) Frame subtraction images for the first 4 trials and the last 4 trials (trials 21–24) in response to the 100 mV depolarization pulse. The internal signal area is somewhat smaller in the last group of 4 trials. (**D**) The optical traces of internal and external signals for the six sets of four trials are shown. Each trace is the average of 4 trials without temporal filtering. Images were recorded at a frame rate of 500 fps. The size bar represents 10 μm.

**Figure 5:**
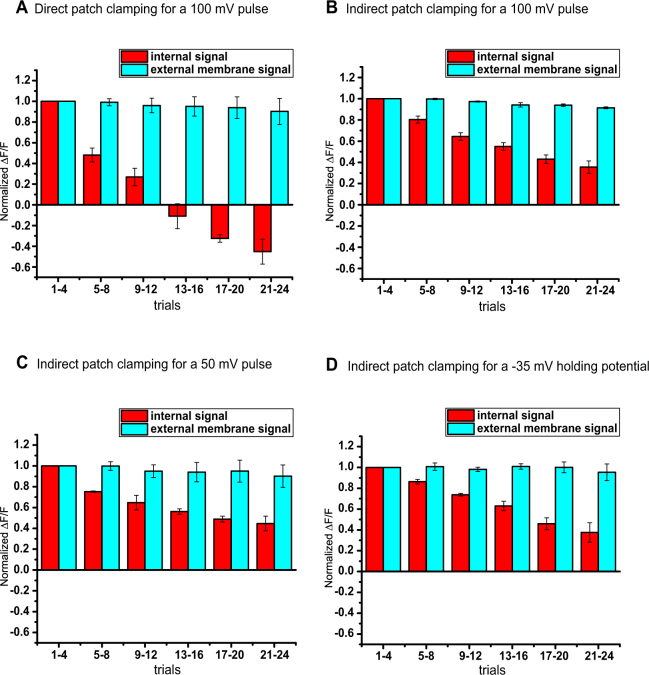
The signal size during repeated patch clamp measurements. (**A**) Normalized internal and external membrane optical signal size for different trials in response to the 100 mV step. HEK239 cells expressing Aahn were voltage-clamped directly. (**B**) Normalized internal and external membrane optical signal size in response to the 100 mV step. HEK239 cells expressing Aahn were voltage-clamped indirectly. (**C**) Normalized internal and external membrane optical signal size in response to 50 mV steps. HEK239 cells expressing Aahn were voltage-clamped indirectly. (**D**) Normalized internal and external membrane optical signal size in response to the 100 mV step with a −35 mV holding potential. HEK239 cells expressing Aahn were voltage-clamped indirectly. The error bars represent the standard error of the mean (n = 3 cells for each plot).

Subtraction of fluorescence intensities of frames prior to a voltage step from the intensities of the frames during the voltage step (black bars in Fig. 1E,H) results in an image identifying the location of the optical signals. We presume that at each pixel the signal is the summation of the internal signal and the external signal. Near the edge of the cell, the external signal should predominate. In the center of the cell, an internal signal will be detected if it is larger than the external membrane signal at that pixel. In Fig. 1D,G, the white area indicates regions where the internal membrane signal predominates while the black areas represent areas where the external signal predominates. We name the reversed signals as “internal signals”. The internal signal is likely obscured by the external signal in other locations. The fluorescent intensity decrease at the edge of the cell during depolarizations (dark areas in Fig. 1D,G) is typical for ArcLight. In this and subsequent figures the blue trace indicates the plasma membrane signal and the red trace indicates the internal membrane(s) signal. The results illustrated in Figs 2–5, were carried out in HEK293 cells.

#### Overlap of the internal signal with the nucleus

Additional results from the HEK293 cell illustrated in Fig. 1 are shown in Fig. 2. In the experiment illustrated in Fig. 2A–E, HEK293 cells were co-transfected with a red fluorescent marker targeted to the nucleus (nls-mCherry)^16^ together with Aahn. The neighbor cell was voltage clamped and subjected to negative and positive voltage steps. The Resting Light Intensity (RLI) of this cell shows high intracellular expression (Fig. 2A). There is no obvious overlap between the internal signal area and the nucleus marker (Fig. 2D). Either a nuclear envelope optical signal is obscured by the plasma membrane signal, or Aahn is not present in the nuclear membranes, or the nuclear membrane does not experience a voltage change. One frame of the 3 dimensional visualization of the frame subtraction during the 100 mV depolarization pulse is shown in Fig. 2E.

#### Overlap with the ER

Co-localization was observed in HEK293 cells expressing both Aahn and an ER marker, Sec. 61, fused to a red fluorescent protein, mCherry^17^ (Fig. 3). As shown in Fig. 3A, a neighbor cell was voltage clamped and stepped with negative and positive voltages. Figure 3B shows high internal fluorescence with some overlap with Sec. 61-beta fused to the fluorescent protein, mCherry, as an ER marker (Fig. 3C). Figure 3D shows evidence of the Aahn internal membrane response (white) and the external membrane response (black) to the100 mV depolarization pulse. Figure 3E shows one frame of the three dimensional visualization of the frame subtraction during the 100 mV step. The overlap of the internal signal with the ER is not perfect, likely due to the fact that the white region only indicates the locations where the internal signal is larger than the external signal. None-the-less, the result shows that the ER is a possible source of the internal signal. Additional examples are shown in Supplemental Fig. S1.

#### Confocal imaging

HEK293 cells co-expressing Aahn with the ER marker (mCherry-Sec. 61-beta) were imaged with a confocal fluorescence microscope to obtain higher resolution images of the overlap. The ER marker overlaps with the internal Aahn expression when Aahn’s internal expression is high (Supplemental Fig. S2). These results suggests that the ER is a possible source of the Aahn internal signal. Cells with limited Aahn internal expression were usually not selected for patch clamp-fluorometry recordings.

#### Comparison of external and internal time courses

Using single and double exponential fitting of the Aahn response to 100 mV steps we estimated the external and internal signal time constants for five cells avoiding pixels near the border between the reversed internal and external signals (Supplemental Table S1). The averaged response from three pixels which had the largest signal was used for both internal and external signals. The average difference in time constants between external and internal signals was small and not significantly different (p = 0.78) (Supplemental Table S1). However, small differences in the time courses of the internal and external signals sometimes resulted in unusual signals at the border between the external and internal regions (Supplemental Fig. S3). The unusual signals are most noticeable during larger depolarizations. Unusual signals at the boundary between internal and external signals were seen in 6 out of 9 HEK293 cells. Supplemental Fig. S4 shows the relative change in fluorescence of the internal and plasma membrane signals as a function of membrane potential. The external and internal signals have very similar voltage dependences.

#### Effect of repeated trials

The internal signal decreased in size during repeated patch clamp measurements while the external signal remained unchanged (Fig. 4). We envision two causes for the decrease in the internal signal: (1) The internal membrane(s) resistance decreases during patch clamp measurements and/or (2) The connection between the internal membrane(s) and plasma membrane is dissipating. The rate of internal signal decrease for indirect patch clamping is smaller than for direct patch clamping (Fig. 5A,B). Using only −50 mv and +50 mv voltage pulses (Fig. 5C) or a more depolarized holding potential (Fig. 5D) did not change the rate of decrease of the internal signal. The internal signal area also shrinks during repeated patch clamp measurements. This internal signal area change is likely due the internal signal size reduction. Since the external signal size remains unchanged and the internal signal size decreases, the area with reversed signal will decrease with repeated measurements. It is unlikely that the decrease observed over several repetitions is due to the accumulation of excess charge in the internal membranes since alternating the hyperpolarizing and depolarizing pulses showed a similar result (Supplemental Fig. S5).

## Discussion

We have optically monitored voltage changes in internal membrane(s) and the plasma membrane simultaneously. Using a new genetically encoded voltage indicator, Aahn, we applied the patch clamp technique to HEK293 cells and cultured neurons and found that there is an electrical interaction between the plasma membrane and the internal membrane(s) of both cell types. The ER is both a continuous membrane bound organelle and has components that are closely opposed to and interacting with the external membrane. Thus, it is very likely that the ER contributes to Aahn’s internal signal. The Golgi apparatus is also widespread in the cytoplasm but, in contrast, is not thought to exist as a continuous membrane bound organelle and is not thought to have close oppositions to the external membrane (M. Terasaki, personal communication). Any Golgi contribution to Aahn’s internal signal is likely to be secondary.

The voltage changes in the internal membrane(s) are highly synchronized with the voltage changes in the plasma membrane (Figs 1–4). In a previous report from our lab, we have shown that pH induced optical signals are very slow suggesting that Aahn’s internal signal is voltage dependent^18^. In eukaryotic cells, ER-PM junctions can be as close as 10 nm^19^ and play a role in the movement of ions and lipids^20^. The voltage-gated potassium channel, Kv2.1, has been shown to direct the architecture of the ER to the PM in both HEK cells and neurons^21^. We speculate that these sites of close apposition could couple voltage alterations of the PM to the ER. One mechanism could be via direct fusion of the ER to the plasma membrane. During calcium depletion of the ER, an ER resident protein, STIM1, is activated and binds to a protein in the plasma membrane ORAI^22^. Reports in Jurkat T cells^23^ and in *Xenopus* larvae hair cells^24^ demonstrated that staining the external leaflet of the plasma membrane with the membrane-impermeable styryl dye, FM1-43 resulted in the staining of the lumen of peripheral ER suggesting the possibility for ER-PM fusion. In a second mechanism the close proximity of the ER and PM at these junctions could result in ephaptic coupling of the two membrane potentials^25^. Other potential coupling mechanisms involve tethering proteins identified in yeast which include orthologs of synaptotagmins^26^ and/or the cytoskeleton. The generation of a mainly internal version of Aahn would facilitate experiments designed to elucidate the voltage coupling mechanism.

In addition to the signaling network represented by the second messenger cascade, here we have shown that an internal membrane, potentially the ER, can act like a “cytoplasmic nervous system” which conducts electrical signals for intracellular communication^9^. One advantage of such a direct electrical communication is the high speed of this process.

The following reports might possibly be explained by electrical connections between external and internal membranes. Friedman *et al*.^27^ found that mitochondrial division occurs at positions where ER tubules contact mitochondria in both yeast and mammalian cells. Caldieri *et al*.^28^ showed that epidermal growth factor receptor endocytosis relies on ER-PM contact sites and local Ca^(2+)^ signaling. ER-PM contact sites have also been implicated in regulating recycling of potassium channels^29^.

The expression pattern of Aahn is also interesting in that accumulation of intracellular expression does not seem to inhibit trafficking to the plasma membrane. Aahn clearly expresses well at the plasma membrane. The voltage-dependent optical signal is quite robust (Figs 1E,H,G,D, 3F and 4D). Indeed, the plasma membrane expression is a potential problem since the reverse polarity of the optical signal at the plasma membrane likely masks the internal signal accounting for our ability to only detect an internal signal in 20% of transfected cells.

Using GEVIs to monitor internal membrane potential changes may help to understand the regulation of the organelle interactome^30^ and improve our understanding of how the cell responds to its environment and stress. We expect that this approach can be used to enhance our view of the role of the ER and other internal organelles in cell physiology both in health and disease.

## Materials and Methods

### Plasmid DNA designs and construction

Introducing the double mutant (P140S and G141D) in the *Ciona* voltage-sensing domain in ArcLight^14^ the new ArcLight-derived probe, Aahn, was generated. G141D was introduced in ArcLight to make ArcLight the same as CC1 at position 141. Primers used for amplification of the first transmembrane domain (S1) which consists the double mutant (P140S and G141D) in first step PCR reaction were: LC226: 5-ATA CGA CTC ACT ATA GGG-3 and LC203: 5-ACTTTTatcGGAAAGACTGAG-3. Primers used for amplification of the S2, S3 and S4 transmembrane domains and florescence protein which consists the double mutant (P140S and G141D) in first step PCR reaction were: LC202: 5-CTC AGT CTT TCC gat AAA AGT-3 and LC186: 5-TCTTTCCTGTACATAACC-3. In the second step PCR, we used primers LC226 and LC186 and combined the first step PCR products. The second step PCR product then was digested with restriction enzymes Nhe1 and BamH1 and inserted into the corresponding sites of the ArcLight construct. The construct, nls-mCherry was a gift from Jinhyun Kim (Addgene plasmid #34911)^16^. DNA sequences for all of the constructs were confirmed by DNA sequence analysis using the dye-termination method (Cosmogenetech).

### Cell culture

HEK293 cells were maintained in DMEM (High Glucose DMEM; Gibco) supplemented with 10% (v/v) fetal bovine serum (FBS; Invitrogen). HEK293 cells were plated onto #0 coverslips coated with poly-L-lysine (Sigma) in a 24-well culture dish and kept in an incubator at 37 °C under air with 5% CO2. Transfection was performed by using Lipofectamine 2000 (Invitrogen) according to the instructions of the manufacturer. Hippocampal neurons were isolated from E18 mouse embryos and maintained in Neuro basal medium with 0.5 mM Glutamax-I and 1 ml of B-27 supplement (Invitrogen) per 50 ml of cultured medium. Four to seven days after cell isolation, transient transfection was accomplished by using 1 to 2 µg DNA per 12 mm coverslip in a 24-well plate by using Lipofectamine 2000 (1 µl per 12 mm coverslip). The neurons were used for experiments 1–3 days after transfection.

### Patch clamp

Voltage clamp recordings were performed in a perfused chamber with the bath temperature kept at 33 °C by a temperature controller and bath solution containing: 150 mM NaCl, 4 mM KCl, 2 mM CaCl2, 1 mM MgCl2, 5 mM D-glucose, and 5 mM HEPES, pH7.4. We used glass patch pipettes (capillary tubing with 1.5/0.84 mm; World Precision Instruments) that were pulled by a P-97 micropipette puller (Sutter Instruments). Patch electrodes had resistances of 3–5 MΩ when filled with intracellular solution containing (in mM) 120 K-aspartate, 4 NaCl, 4 MgCl2, 1 CaCl2, 10 EGTA, 3 Na2ATP, and 5 HEPES, pH 7.2. Voltage-clamp recordings in the whole-cell configuration were performed using a Patch Clamp EPC10 amplifier (HEKA) with a holding potential of −70 mV except for the experiment of Fig. 5D where the holding potential was −35 mv.

### Wide-field imaging

Whole-cell patch clamped cells were imaged with an OlympusIX71 microscope with a 60 × 1.35 numerical aperture oil-immersion lens (Olympus). A 75 W xenon arc lamp (Cairn Research) was used as the excitation light source. The excitation filter for the green fluorescence protein (Aahn) was 472/30, the emission filter was 496/LP and the dichroic was 495 (Semrock, NY). The excitation filter for the red fluorescence protein (mCherry-NLS) was 562/40, the emission filter was 641/75 and the dichroic was 593 (Semrock, NY). The fluorescence image was demagnified by an Optem zoom system, A45699 (Qioptiq LINOS) and projected onto the 80 × 80 pixel chip of a NeuroCCD-SM camera controlled by NeuroPlex software (RedShirtImaging, GA). The images were recorded at a frame rate of 500 fps. The bright field images were demagnified by an Optem zoom system, A45699 (QioptiqLINOS) and captured by a (Hitachi, Tokyo) CCD camera (KP-D20BU). The frame grabber (PCI-RTV24) and its corresponding software (ADLINK ViewCreatorPro) was used to control the Hitachi camera and record the images.

### Confocal Imaging

Using a Plan-Apo 60×/1.40 Oil DIC N2 objective and a Nikon Eclipse Ti (Nikon, Japan) confocal laser scanning microscope, confocal images were obtained. A 488 nm wavelength laser was used for Aahn excitation. The emission filter for the Aahn was 500–550 nm. A 561 nm wavelength laser was used for ER marker (mCherry-Sec. 61-beta) excitation. The emission filter for these red florescent proteins was 570–620 nm. NIS-Elements microscope imaging software was used for image acquisition and processing.

### Optical signal analysis

We used NeuroPlex software (RedShirtImaging) to calculate the $\% \Delta F/F$, the dark image was subtracted from all frames, then the average of a region of interest in each frame $(F)$ is subtracted from the average of the region taken from ten frames prior to the event of interest $({F}_{0})$ and then this value was divided by ${F}_{0}$, i.e. $\% {\rm{\Delta }}F/F=(\frac{F-{F}_{0}}{{F}_{0}})100$. The optical traces were imported into Origin 8.6 (OriginLab, MA) for the analysis of response time constants, ${t}_{1}$ and ${t}_{2}$. The probe dynamics were fit with either a single exponential equation $[y={A}_{1}{e}^{(-X/{t}_{1})}+{y}_{0}]$ or a double exponential equation $[y={y}_{0}+{A}_{1}{e}^{(-(X-{X}_{0})/{t}_{1})}+{A}_{2}{e}^{(-(X-{X}_{0})/{t}_{2})}]$ where ${A}_{1}\,$and ${A}_{2}\,$are amplitudes, and ${t}_{1}\,$and ${t}_{2}$ are time constants in ms.

## Electronic supplementary material

Supplementary materials
