# Supplementary material for: Monitoring voltage fluctuations of intracellular membranes
Source: Sci Rep. 2018 May 2;8:6911. doi: 10.1038/s41598-018-25083-7 (PMC5932030; doi:10.1038/s41598-018-25083-7)
Supplement: Supplementary file 1 — Supplementary materials [file 41598_2018_25083_MOESM1_ESM.doc]

Supplementary Materials:

**Title: Monitoring voltage fluctuations of intracellular membranes**

**Authors:** Masoud Sepehri Rad^1^, Lawrence B. Cohen^1,2,*^, Oliver Braubach^1^, Bradley J. Baker^1,3,*^

**Affiliations:**

^1^Center for Functional Connectomics, Brain Science Institute, Korea Institute of Science and Technology (KIST), Seoul, 02792, Korea.

^2^Department of Cellular and Molecular Physiology, Yale University School of Medicine, New Haven, CT 06520, USA.

^3^Department of Neuroscience, Korea University of Science and Technology, Daejeon, South Korea.

*Correspondence to: masoud.sepehri15@yahoo.com, lawrence.b.cohen@hotmail.com and Bradley.baker19@gmail.com.


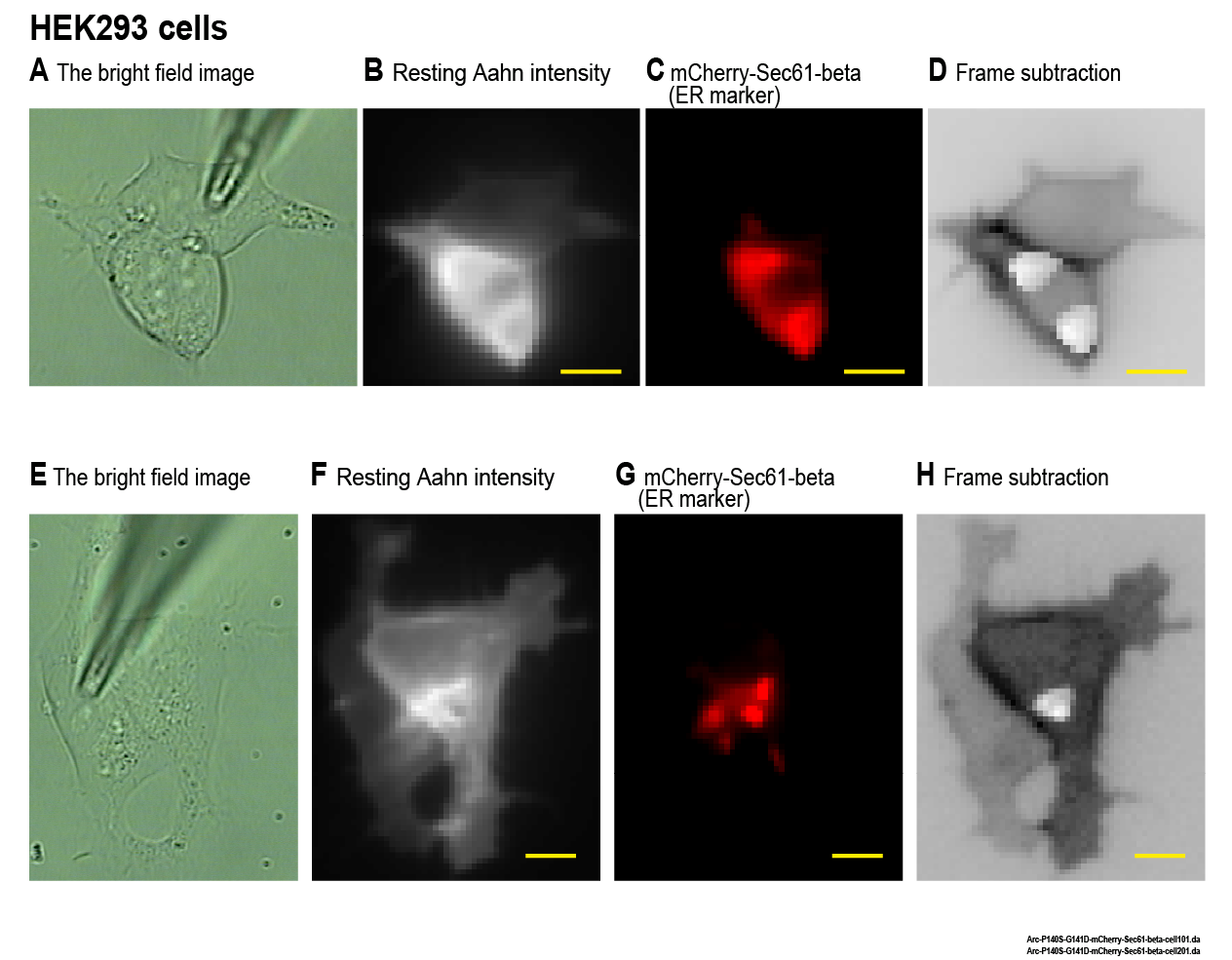


**Figure S1.** **Two additional examples for the overlap between the ER marker and the internal signal area in HEK293 cells.** HEK 293 cells expressing Aahn and mCherry-Sec61-beta were voltage-clamped. The data for two examples are shown in (**A** to **D** and **E** to **H**) for similar experiments as illustrated in Fig. 3. There is overlap between the internal signal area and the ER marker. Size bar represents 10 μm.

**
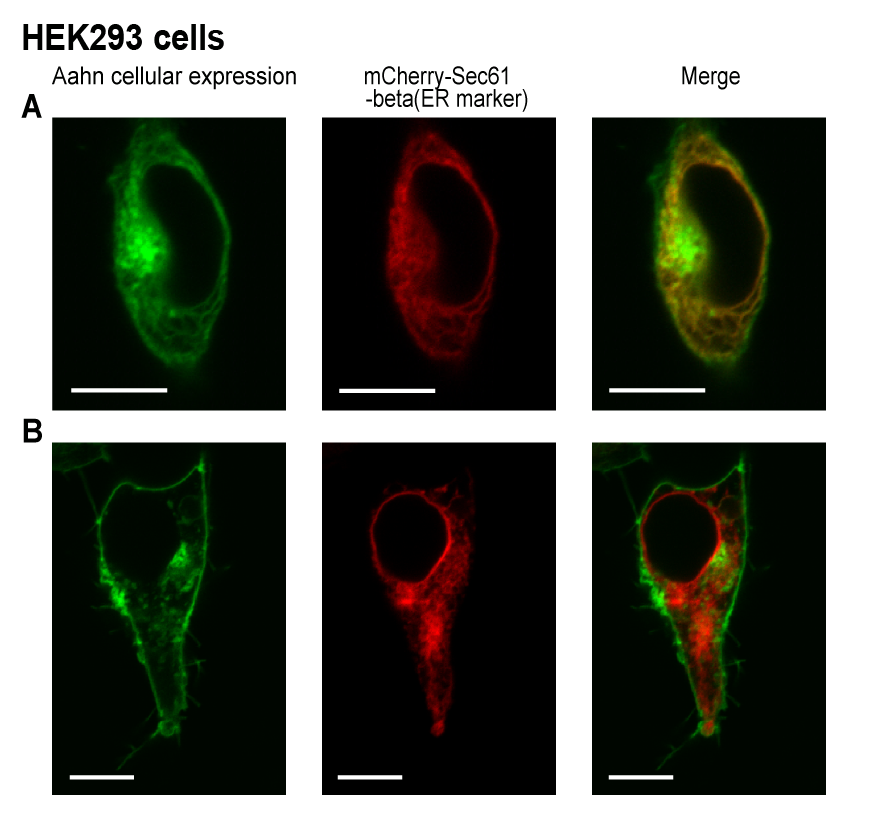
**

**Figure S2.** **The overlap between the ER marker and the Golgi marker with internal Aahn expression using confocal microscopy.** (**A** and **B**) HEK293 cells co-expressing Aahn and a red florescence protein (mCherry-Sec61-beta) were imaged with a confocal fluorescence microscope. Size bars represent 5 μm.

**
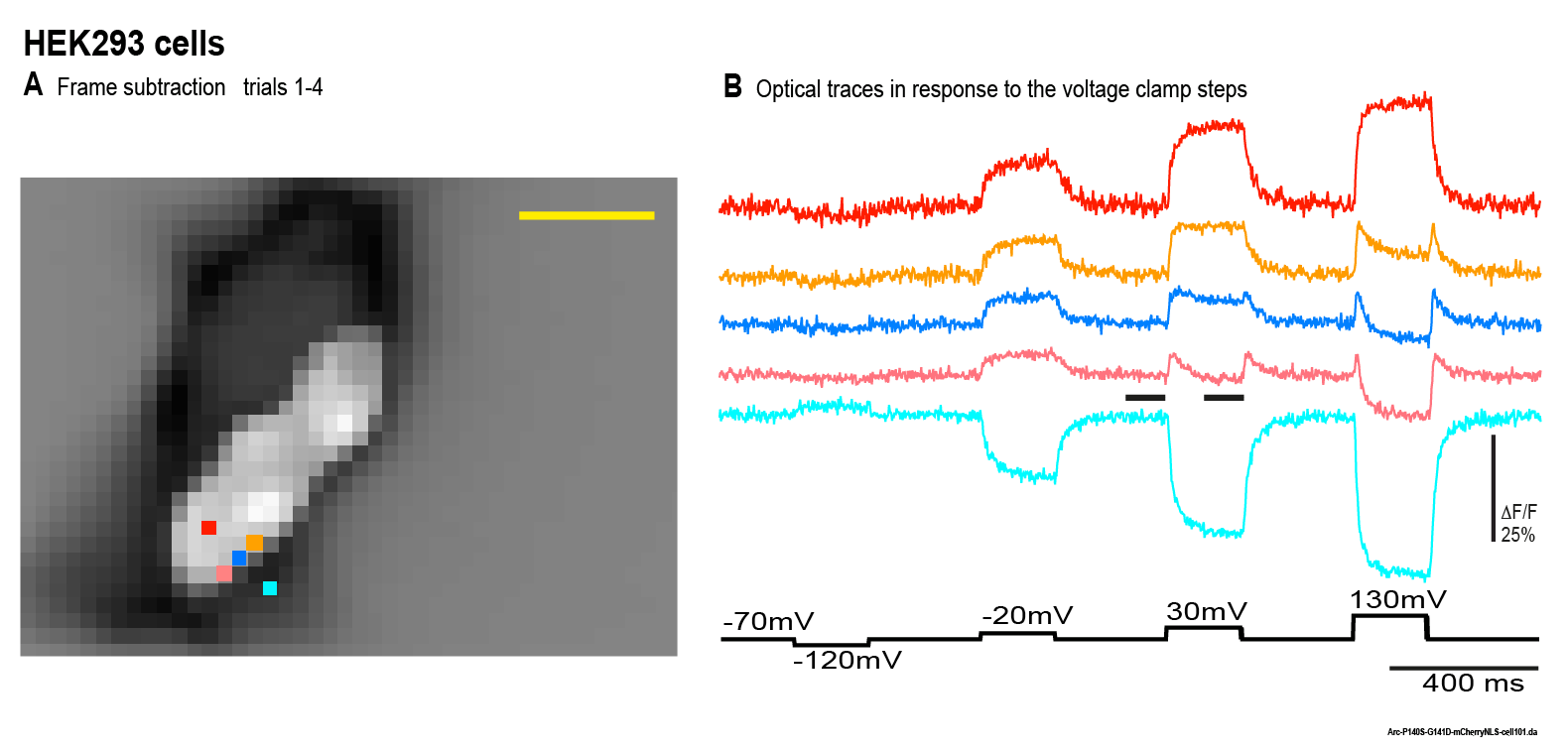
**

**Figure S3. The internal and external optical signals have slightly different time courses in HEK293 cells.** HEK 293 cells expressing Aahn and nls-mCherry were voltage-clamped. (**A**) The frame subtraction image of the HEK293 cell in response to the 100 mV depolarization pulse for the first 4 trials from the same data as shown in Fig. 1A-F. (**B**) The red trace indicates the internal signal, blue trace shows the plasma membrane signal. The different time course of the internal and external optical signals results in unusual traces from pixels at the border (brown, dark blue and pink traces). All traces are shown without temporal filtering. Images were recorded at a frame rate of 500 fps. The size bar represents 10 μm.


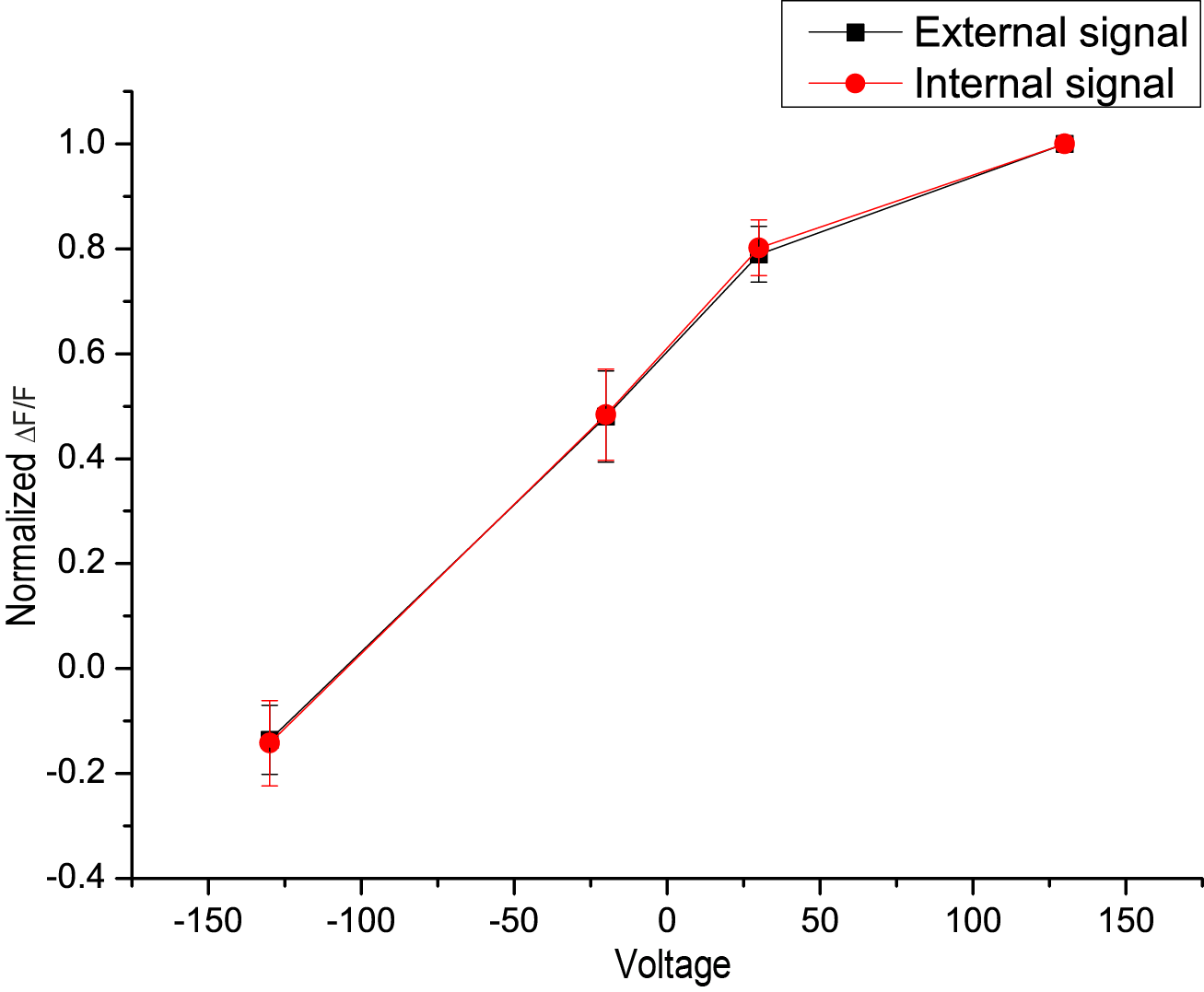


**Figure S4. Voltage sensitivity of the internal and the external optical signals in HEK293 cells.** The external and internal signals have similar voltage sensitivity. For a better comparison we have normalized both the external and the internal signals. The external signal has also been inverted.

**
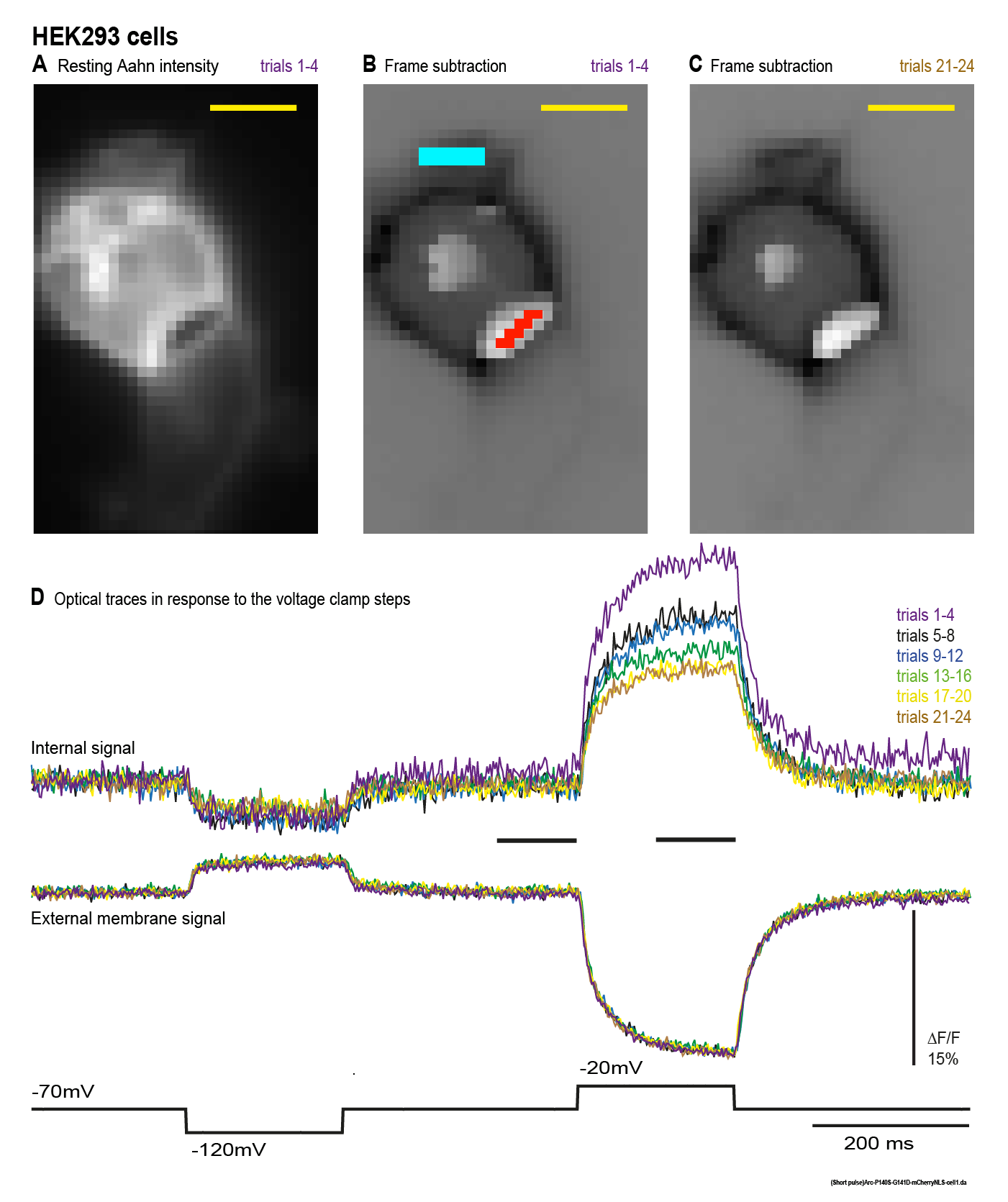
**

**Figure S5. The internal optical signal size decreases during repeated patch clamp measurements for ±50 mV steps in HEK293 cells.** A HEK 293 cell expressing Aahn was voltage-clamped. (**A**) The resting light image of the Aahn expression. (**B** and **C**) Frame subtraction images for the first 4 trials and the last 4 trials (trials 21-24) in response to the 50 mV depolarization pulse. (**D**) The optical traces of internal and external signals for six sets of four trials are shown. Each trace is the average of 4 trials without temporal filtering. Images were recorded at a frame rate of 500 fps. The size bar represents 10 μm.

**Table S1.** **The time course difference between internal and external signals.** The internal and external optical signals have slightly different time courses in HEK293 cells. Using single and double exponential fitting of 100 mV responses, the time constants were calculated for both direct and indirect patch clamp measurements.
